# Incidents of Animal Life

**Published:** 1887-12-31

**Authors:** F. O. Morris

**Affiliations:** Author of "A History of British Birds," "The Gamekeepers' Museum," and the Humanity Series of School Books


### Incidents of Animal Life.

Collated by the Rev. F. O. Morris, B.A., Author of "A History of British Birds," "The Gamekeepers' Museum," and the Humanity Series of School Books.

> "He prayeth best, who loveth best
> All things both great and small;
> For the dear God who loveth us,
> He made and loveth all."—Coleridge.

### A Cat Story.

Your interesting letter about "The Cat and the Swallows" reminds me of a cat I had some years ago whilst living at Kritlington in Oxfordshire. This cat was constantly in the room whilst we were at dinner, and, when wanting to leave the room, would jump on to a chair near the fireplace, lay hold of the rope with its two paws and ring the bell, then jump down, walk to the door, and wait until the servant came to inquire what was wanted, when it would go quietly away as if it had had nothing to do with it. This I had seen the cat do several times; and one evening when two gentlemen were dining with me I drew their attention to what I thought the cat was going to do, and the same process was repeated. The gentlemen would not have believed it, they said, unless they had seen it, nor should I. This same cat was a great poacher, and constantly brought home hares and rabbits, and one evening jumped through a window four or five feet from the ground with a young leveret in its mouth bigger than itself, and laid it on the mat outside the drawing-room door.

### Rats and their Ways.

Your account, related some days ago, about a rat and eggs was read aloud by me to my family, and with one accord they wished me to write you of a somewhat similar experience of my own. Some years ago I bred poultry to a large extent, and was annoyed to find day by day no eggs from certain hens I knew were laying, and who from appearances ought not to be eating their eggs (a disease prevalent among hens not properly cared for). One day one of my children came running in and begged me to come out quickly and quietly, and see the thieves. I was much astonished to find three rats—one on his back, one pulling him along, and one keeping him steady, so I supposed was the duty of the last one, for the rat on his back very rarely lost his equilibrium. The centre rat had his four legs encircling an egg. I left them undisturbed, and in a very short time—about a minute and a half—they had safely conveyed the egg from the hen-house into the stables, when I had just time to run in and secure it as it was being drawn into one of their holes between the stalls. The place this happened at was Helensburgh, where some neighbours kept poultry also; all the nests were in divisions about a foot or fifteen inches from the ground, with no ladder: it was simply a hop for the brahmas to get up. How the rats got the egg down I cannot imagine, for even by the process described it would be difficult, unless the rat clasping the egg was gradually lowered down, the tail acting as a rope. If you will read La Fontaine's first fable, 10th Book, if you are not already acquainted with it, you will, I think, be interested, as it shows that the ways of rats with eggs were well known in his day, and how much further back one cannot say. I should not be surprised to find something about it either in Montaigne or Rabelais. The edition of La Fontaine now before me is beautifully illustrated by Granville, and this very action of two rats is the subject of a most amusing woodcut. The draught-rat is trudging along with the tail of his comrade over his shoulders, the latter lying on his back with the eggs clasped to his belly by his four paws. They look for all the world like two costermongers—serious, busy, and resourceful. Another trick of rats is to suck out the contents of a bottle of olive-oil, and this was also seen by the same interested observer. The cork is gnawed off, and the sagacious animals insert their tails, and so easily get out the liquid.

### Two Dogs and some Rabbits.

"A curious story of canine sagacity is reported in the Cologne journals. The owner of a number of rabbits near Barnicu found that for six successive nights one of his rabbits was stolen from the house which he had made for them out of a wooden case, which stood a few inches above the ground. At the top of it an opening had been made, about the width of two hands, which was closed at night by a board on which heavy stones were laid. The house having been thus secured, and as it was found each morning that only one rabbit had been stolen, and that all the rest were quite uninjured, it was considered impossible for a weasel to have effected the theft. It was therefore supposed that human hands had been at work. The owner consequently first made the opening more secure by nailing down one side of the board, and covering it with grass and stones, and then hid himself in order to watch for the thief. At one o'clock in the morning he heard a noise at the rabbit-house, and was not a little astonished to see two dogs instead of a man on the top of it. One was a large dog of the neighbourhood, well known to him, a cross between a St. Bernard and a large woolly collie, feared by all other dogs; the second was a stranger, a small terrier, just slender enough to get through the hole into the rabbit-house. The big dog, who on other occasions never noticed his smaller comrades, had evidently come to an understanding with his little friend about the nocturnal rendezvous. The big dog scratched away all the grass and the stones, dragged up the board, and let the terrier jump through the hole. The latter returned in a few minutes with a rabbit in his mouth, which he presented to his great friend, and both proceeded to devour their supper undisturbed."

---

REMARKABLE SAGACITY OF A DOG.

At Cardiff, Captain Thompson, of the steamer *Muley Hassan*, was presented with a silver medal for saving life. The steamer was passing through the Straits of Gibraltar, when the captain's retriever showed great signs of restlessness, and eventually jumped overboard. A boat was lowered, and the dog was discovered holding the collar of the coat of a drowning man lying across two oars. He was afterwards discovered to be the only surviver of a Spanish revenue felucca, which had been upset four hours previously.

---

HOW A HORSE SAVED HIS OWN LIFE.

A poor horse, suffering under severe colic, was brought to a Mr. Seton, of Lochlane, who was skilled in horseflesh; he set so actively about his business that the horse was soon sent home completely relieved from his complaints. Some weeks after the horse again suffered from a fresh attack of the same complaint, but as it was not convenient to remove him to the smithy he was allowed to remain in the stable. In the morning, by some means he disengaged his neck from the collar which fastened him, and set off for his old friend the smith at Lochlane, two miles away. Being rather early, he forced open the door of the smithy, and took his station exactly on the same spot he formerly stood. Mr. Seton in due time made his appearance, and soon perceiving his patient enduring the gripes and agonies of colic, at once administered to his relief. The owner had scarce time to extend his search to the smithy, when he found his steed safe and sound, ready to carry him home.

---

THREE INSTANCES OF CANINE INSTINCT.

The Rev. F. E. Gretton writes: I recently read a paper of yours on "Dogs and their Doings," which called to my remembrance three instances of canine sagacity in days long gone by, that may possibly have interest with you.

1. Early in this century the then Duke of Buccleuch sent two couples of hounds to Lord Lonsdale. The dogs were sent on their travels *by sea* from Leith, and duly reached the Cottesmore kennels. The first day that they were taken out with the pack the run ceased at Dushhorn, close to the Great Northern Road, and when the hounds were collected, one of the whips said: "Where are the Scotchmen?" They had in fact levanted, and no trace could be found of them, till, weeks after, news came that, sore-footed and half-starved, they had arrived at Dalkeith. Pray, is the line between instinct (as it is called) and reason real or imaginary?

2. Very many years ago a brother of mine, resident at Upton Bishop, rode over to visit us at Hereford. A sudden flood in the Wye blocked his journey home. He had brought with him Bruno, a favourite pointer dog—very clever, quickish in temper, and a sorry thief. Detected in pilfering, he was rated by one of the servants—possibly swished. However, he made himself scarce forthwith, and when, nearly a week afterwards, his master returned home, he found Bruno had arrived some days. But how did he get there? That he returned by the road he came was simply impossible, the waters were out so far. Moreover, there was evidence that he had made his way through Ross, for a friend of my brother's saw him running very wildly, and as if lost, through the town. Now, Bruno very well knew the road from Upton to Hereford and Upton to Ross, but the road from Hereford to Ross he had never seen in his life. How came he to choose it?

3. I once had a large flea-bitten dog—whence his name, Flea—who, not being exclusive in his tastes, formed a strong intimacy with a pig. They were shut in the yard, between which and an outer court was a rather heavy gate, which Flea was not strong enough to pull back, while the latch was too high for the pig to reach. How, after a cabinet council, did they effect their escape? Flea stood on his hind legs, and with his nose lifted the latch, while piggy put his snout under the door—too heavy for Flea—drew it back, and both triumphantly marched out! I saw them do it.

